# Synergistic Effects of Maize Growth and Soil Respiration on Yield Under Tillage Regime Conversion

**DOI:** 10.3390/plants15203134

**Published:** 2026-10-14

**Authors:** Jingkun Lu, Tianhao Luan, Nan Wang, Chen Xu, Haoran Liu, Hongjun Wang, Baoyu Chen

**Affiliations:** Jilin Academy of Agricultural Sciences (Northeast Agricultural Research Center of China), Changchun 130033, China; lujingkun5258@163.com (J.L.); luantianhao@jaas.com.cn (T.L.); wangnan0307@jaas.com.cn (N.W.); xuchen@jaas.com.cn (C.X.); lhr337@126.com (H.L.)

**Keywords:** soil respiration, yield, temperature sensitivity, tillage practice transition, synergistic effect, maize (Zea mays) growth

## Abstract

Soil respiration is a major pathway of carbon loss from agricultural soils and is substantially influenced by tillage practices. However, the short-term effects of different tillage system transitions on soil respiration and maize yield remain unclear. A field experiment was therefore conducted with five treatments: conventional tillage–conventional tillage (CK), mulching–mulching (CS), conventional tillage–rotary tillage (NR), rotary tillage–rotary tillage (RS), and mulching–rotary tillage (CRS). Soil respiration, maize growth, and yield were monitored throughout the growing season. The results showed that tillage transition significantly altered the soil respiration rate and its component structure. Higher soil respiration increased CO_2_ release, raised canopy CO_2_ concentration, and accelerated dry matter accumulation. The RS exhibited the highest soil respiration, with a peak rate of 16.83 μmol m^−2^ s^−1^, of which root respiration accounted for 64.16%, and improved soil porosity and promoted aboveground biomass accumulation. The CRS treatment achieved the highest leaf area index, dry matter accumulation, and yield, representing a 51.49% increase over the control, and had the lowest Q_10_ value (2.97), indicating that respiration under this treatment was more regulated by substrate availability than by temperature. The CS treatment minimized carbon loss while maintaining grain yield. Overall, tillage transitions that improve soil aeration and root activity were generally associated with higher maize yields, but different pathways created distinct trade-offs among respiration, carbon turnover, and harvestable yield. These findings suggest that conservation tillage transitions have considerable potential for balancing yield maintenance with soil respiration regulation, providing useful guidance for sustainable high-yield maize production in semi-arid regions.

## 1. Introduction

Soil respiration is the primary biological process by which soil releases CO_2_ into the atmosphere [1]. It comprises autotrophic respiration, mainly from plant roots and their associated microorganisms, and heterotrophic respiration, which results from the microbial decomposition of organic matter [2]. This process represents a critical link between plant photosynthesis and soil carbon pools, and even small changes in soil respiration can substantially affect atmospheric CO_2_ concentrations [3]. Among the major management factors, tillage intensity and straw management regimes exert pronounced control over soil CO_2_ efflux. By altering soil physical structure, chemical properties, microbial activity, and root growth dynamics, these practices regulate both the rate of soil respiration and the relative contributions of its components. For example, conventional tillage generally disrupts soil structure, accelerates organic matter decomposition, and increases heterotrophic respiration, whereas conservation tillage practices, such as no-tillage and straw mulching, help maintain soil stability and may alter the proportion of autotrophic to heterotrophic respiration [4].

Numerous studies have examined the effects of different tillage practices, such as conventional tillage, rotary tillage, and no-tillage, as well as straw return methods, on soil respiration [5]. However, the findings have been inconsistent, likely because of differences in climatic conditions, soil types, cropping systems, and research methodologies [6,7]. More importantly, most previous studies have focused on long-term, static tillage systems, while overlooking the dynamic nature of actual agricultural production. For a long time, conventional tillage practices, particularly rotary tillage with straw removal, have been widely adopted by farmers. Although these practices facilitate seedbed preparation and short-term moisture regulation to some extent, they also intensify wind and water erosion, accelerate the mineralization and decomposition of soil organic carbon, and consequently weaken the stability of the soil carbon pool. In recent years, conservation tillage practices, such as straw mulching, have been promoted in some areas [8]. However, in actual agricultural production, dynamic interannual transitions in tillage practices are common due to considerations such as machinery availability, cost, and pest or weed control [9]. For example, farmers may shift from protective straw mulching to more disruptive rotary tillage. Compared with fixed tillage systems, such short-term transitions may impose more complex and distinct disturbance effects on soil physical structure, microbial community activity, and carbon cycling processes [10]. Nevertheless, research on how dynamic tillage transitions affect soil respiration and its components, including autotrophic and heterotrophic respiration, remains limited. In addition, temperature sensitivity (Q_10_) is a key parameter for assessing the strength of soil respiration feedback to climate warming [11]. How tillage transitions influence the Q_10_ values of different respiration components therefore remains an important question.

Tillage practices also exert strong effects on crop productivity. Conservation tillage measures, such as no-tillage and straw mulching, can improve soil water storage, reduce erosion, and increase soil organic matter, thereby supporting crop growth and yield stability [12,13]. Straw return can enhance soil fertility and water availability; however, excessive straw mulching may delay seedling emergence and reduce stand establishment. In addition, short-term tillage transitions may have more pronounced effects on crop growth than long-term, stable tillage systems, because crops must adapt to sudden changes in soil physical conditions and nutrient supply [14]. The experimental site, Baicheng City in western Jilin Province, is a typical semi-arid agricultural region in Northeast China, where low temperatures, aridity, and concentrated precipitation make soil respiration dynamics especially sensitive to tillage disturbance. Moreover, as an important grain-producing area in Jilin Province, Baicheng plays a critical role in regional food security and agricultural sustainability. Therefore, investigating how dynamic tillage transitions affect soil respiration and its components in this semi-arid region is of great significance for optimizing tillage management, enhancing soil carbon sequestration, and promoting sustainable agricultural development under climate change.

In this context, the present study investigated the integrated effects of annual dynamic tillage transitions on soil respiration and yield under straw return conditions. Specifically, the objectives were to (1) quantify the changes in soil respiration rate and the contribution proportion of root respiration under different tillage system transformation measures; (2) clarify the effects of different treatments on crop growth and yield; and (3) explore the synergistic effects of maize growth and soil respiration on yield under tillage system transitions. The outcomes of this work are intended to inform site-specific tillage adjustments that simultaneously support carbon sequestration and mitigate emissions from agricultural soils.

## 2. Materials and Methods

### 2.1. Overview of the Experimental Site

The experimental site is located at the Experimental Station of the Jilin Academy of Agricultural Sciences, Qingshan Town, Taobei District, Baicheng City, Jilin Province, China (45°688′ N, 122°856′ E). The region has a continental monsoon climate, characterized by four distinct seasons, relatively abundant heat, water, and light resources, and a coordinated water–heat regime. During the crop growing season from May to September, precipitation was 355.6 mm, and the annual average temperature was 5.2 °C. The average number of days with wind force greater than level 8 was 18.1, and strong winds persisted for extended periods in spring. The average frost-free period was 144 days, and the annual average sunshine duration was 2915 h.

### 2.2. Experimental Design and Sample Collection

According to the USDA soil texture classification, the main soil type at the experimental site is loam [15]. Before the experiment was initiated, soil samples were collected from the 0–20 cm surface layer using a soil auger. The basic physicochemical properties of the soil are presented in Table 1.

The experimental site was established in 2009 and was designed to include three representative tillage practices: (1) Conventional tillage without straw: in this treatment, straw was removed from the field by burning. The soil was then tilled using a rotary tiller (1GKNBM-330, YAAO, China), followed by ridging and seeding. (2) No-till with straw mulching: straw was coarsely crushed using a straw shredder. After subsoiling, direct seeding was carried out with a no-till seeder, and the shredded straw was retained on the soil surface as mulch. (3) Rotary tillage with straw incorporation: straw was finely crushed using a straw shredder (1JH-165Q, YTO, China) and then fully incorporated into the soil using a rotary tiller, followed by ridging and seeding. The experimental site and field operations are shown in Figure S1. Although the long-term experimental site was established in 2009, crop yield and yield component data in the present study were collected only during the 2024 growing season, because the objective was to examine the short-term effects of tillage practice changes on soil respiration and crop performance. The 2023 season was used as the previous crop season for comparison of tillage transitions, and the 2024 season served as the current season for data collection. The field experiment was arranged in a randomized complete block design with five treatments and three replications, and each plot had an area of 666 m^2^. The treatments included both continuous treatment scenarios, in which the same practice was applied for two consecutive years, and transition treatment scenarios, in which different practices were applied across the two years. The specific tillage treatments included conventional–conventional (CK), representing continuous conventional tillage in which straw was removed from the field by burning, followed by rotary tillage, ridging, and seeding; mulching–mulching (CS), representing continuous straw mulching with straw incorporation; conventional–rotary tillage (NR), representing a transition from conventional tillage to rotary tillage with straw incorporation; rotary tillage–rotary tillage (RS), representing continuous rotary tillage with straw incorporation; and mulching–rotary tillage (CRS), representing a transition from straw mulching to rotary tillage with straw incorporation. Detailed treatments and their corresponding codes are presented in Table 2.

Under the different tillage treatments, two sampling methods were used to measure soil respiration. For the non-root-zone treatment, PVC collars (20 cm in diameter and 12 cm in height) were inserted into plant-free plots. For the root-zone treatment, PVC collars were placed around root-zone plots. Each treatment was replicated three times. Photographs of the field measurements of the relevant indicators are shown in Figure S2.

The experimental crop was maize, and the cultivar used was “Xiangyu 998”. Sowing was carried out at the end of April each year at a planting density of 70,000 plants ha^−1^. All plots were managed under a uniform fertilization regime: before sowing each year, and in conjunction with the corresponding tillage treatment, compound fertilizer was applied once by deep placement at a rate of 750 kg ha^−1^. The nitrogen–phosphorus–potassium ratio of the applied compound fertilizer was 28-12-14 (N-P_2_O_5_-K_2_O). This fertilization regime ensured consistency in the initial nutrient supply among all treatments.

### 2.3. Soil Physicochemical Analysis

Soil samples collected from the 0–20 cm layer before the experiment were air-dried, ground, and passed through a 2-mm sieve for the analysis of basic physicochemical properties. The specific methods and instruments used for each parameter were as follows. Soil pH was measured in a 1:2.5 (soil–water) suspension using a laboratory pH meter (PHBJ-261L, LEICI, China). Apparent electrical conductivity (ECa) was determined in situ using a portable sensor (Delta-T Devices Ltd, Burwell, Cambridge, UK). Soil organic matter (SOM) was determined by the potassium dichromate (K_2_Cr_2_O_7_) wet oxidation method (Walkley–Black method) and quantified by titration. Available nitrogen (AN) was determined by the alkali hydrolysis diffusion method, which primarily assesses hydrolysable nitrogen. Available phosphorus (AP) was extracted with sodium bicarbonate (Olsen method), and its concentration was determined colorimetrically using a UV/Vis spectrophotometer (Shimadzu UV-1900i, Kyoto, Japan). Available potassium (AK) was extracted with ammonium acetate (NH_4_OAc), and its concentration was measured using a flame photometer (Sherwood Model 410, Cambridge, UK) [16].

### 2.4. Measurement of Soil Respiration Rate, Temperature and Moisture

Soil CO_2_ efflux was measured using an LI-8100A infrared gas analyzer (LI-COR Biosciences, Lincoln, NE, USA). According to the standard protocol, PVC collars (20 cm in diameter and 12 cm in height) were installed 10 cm into the soil at each measurement location 24 h before sampling, leaving 2 cm exposed above the ground surface. Any regrowing vegetation inside the collars was carefully removed before measurement. During sampling, the survey chamber (model 8100-103, LI-COR Biosciences, Lincoln, NE, USA) was securely placed on the collar for measurement. Simultaneously, soil temperature and volumetric water content (VWC) were monitored adjacent to each collar using a 6000-09TC thermocouple probe (METER Group, Inc., Pullman, WA, USA) and an 8100-204 Delta-T Theta soil moisture probe (METER Group, Inc., Pullman, WA, USA), respectively. Both probes were inserted to a depth of 5 cm. Measurements were conducted approximately every 20 days from June to October, covering the entire maize growing season. On each sampling day, measurements were taken between 09:00 and 12:00 local time. Each treatment was replicated three times.

### 2.5. Calculation of Soil CO_2_ Flux

The measured CO_2_ flux, referred to as soil respiration, is denoted as *R_s_* in the subsequent calculations. Soil CO_2_ flux (*F_c_*, expressed in μmol CO_2_ m^−2^ s^−1^) was calculated directly from the rate of CO_2_ concentration increase inside the closed chamber of the LI-8100A system [17]. The instrument uses the following fundamental equation:

(1) $$F_{c}=\frac{10VP_{0}\left(1-\frac{W_{0}}{1000}\right)}{\mathrm{RS}\left(T_{0}+273.15\right)}\frac{\partial C’}{\partial t}$$

where *F_c_* (μmol m^−2^ s^−1^) is the soil CO_2_ emission flux, *V* (cm^3^) is the volume, *P*_0_ is the initial pressure (kPa), *W*_0_ is the initial water vapor mole fraction (mmol mol^−1^), *S* is the measured soil surface area (cm^2^), *T*_0_ is the initial air temperature (°C), and $\frac{\partial C^{’}}{\partial t}$ is the initial rate of change in the H_2_O-corrected CO_2_ mole fraction (μmol mol^−1^ s^−1^).

### 2.6. Calculation Method of Root Respiration

This study employed the root exclusion method to quantify the contribution of root respiration to total soil respiration [18]. Root respiration was calculated as the difference between soil respiration in the root zone (measured with root collars and considered total respiration) and that in the non-root zone (measured with static collars devoid of living roots). The contribution ratio of root respiration (%) was defined as the proportion of root respiration to total soil respiration.

### 2.7. Temperature Sensitivity Analysis of Soil Respiration

The relationship between soil respiration rate (*R_s_*) and soil temperature (*T*) was quantified using the Van’t Hoff exponential model [19]:

(2) *R_S_* = *a*∙*e^bT^*

where *R_s_* is the measured soil respiration rate (μmol m^−2^ s^−1^), *T* is the simultaneous soil temperature at 5 cm depth (°C), *a* is a model parameter representing the base respiration rate at 0 °C, and *b* is the temperature response coefficient. The model parameters (*a* and *b*) were obtained by linear regression of the logarithmically transformed equation:

(3) ln(*R_S_*) = ln(*a*) + *bT*

where *R_s_* is the measured soil respiration rate (μmol m^−2^ s^−1^), *T* is the simultaneous soil temperature at 5 cm depth (°C), *a* is a model parameter representing the base respiration rate at 0 °C, and *b* is the temperature response coefficient. The model parameters (*a* and *b*) were obtained by linear regression of the logarithmically transformed equation:

(4) *Q*_10_ = *e*^10^*^b^*

For each treatment, all paired soil respiration and soil temperature data collected throughout the growing season were pooled to establish the *R_s_*-*T* relationship and to determine a single representative *Q*_10_ value for that treatment.

### 2.8. Leaf Photosynthetic Measurements

At the silking stage, the ear leaves of five representative plants per plot were selected for the measurement of net photosynthetic rate (Pn), stomatal conductance (Gs), intercellular CO_2_ concentration (Ci), canopy CO_2_ concentration (Ca), and transpiration rate (Tr). All measurements were conducted on clear mornings between 09:00 and 11:00 under stable light conditions. An open-flow infrared gas analyzer (LI-6400XT, LI-COR Biosciences, Bourne, MA, USA) was used for all measurements.

### 2.9. Measurement of Plant Growth Parameters and Yield

At key growth stages, representative plants were selected from each treatment for the measurement of agronomic traits. Plant height and stem diameter were measured using a measuring tape and a digital caliper, respectively. Leaf area index (LAI) was determined using a leaf area meter (LAI-2200C, Lincoln, NE, USA). To determine aboveground dry matter, the sampled plants were separated into leaves, stems, and reproductive organs (when present). All plant parts were first oven-dried at 105 °C for 30 min to deactivate enzymes and then dried at 80 °C to constant weight before weighing. At physiological maturity, all maize plants in a 10 m^2^ central area of each plot were manually harvested to minimize border effects.

### 2.10. Statistical Analysis

Statistical analyses were performed using SPSS 25.0 and OriginPro 2023. Figures were generated using GraphPad Prism 10.1 and R. At least three independent replicates were used for data collection and analysis. One-way analysis of variance (ANOVA) was used to compare soil respiration and crop growth performance among tillage treatments, followed by Duncan’s multiple range test for post hoc comparisons. For all analyses, the significance level was set at *p* < 0.05. The interactive effects of temperature and moisture on soil respiration were evaluated using two-way ANOVA in GraphPad Prism. In addition, simple linear regression was used to analyze their individual relationships with soil respiration. Correlations among the measured variables were examined using OriginPro and visualized as correlation heatmaps.

## 3. Results

### 3.1. Crop Growth Performance in Response to Tillage Practices

**Figure 1:**
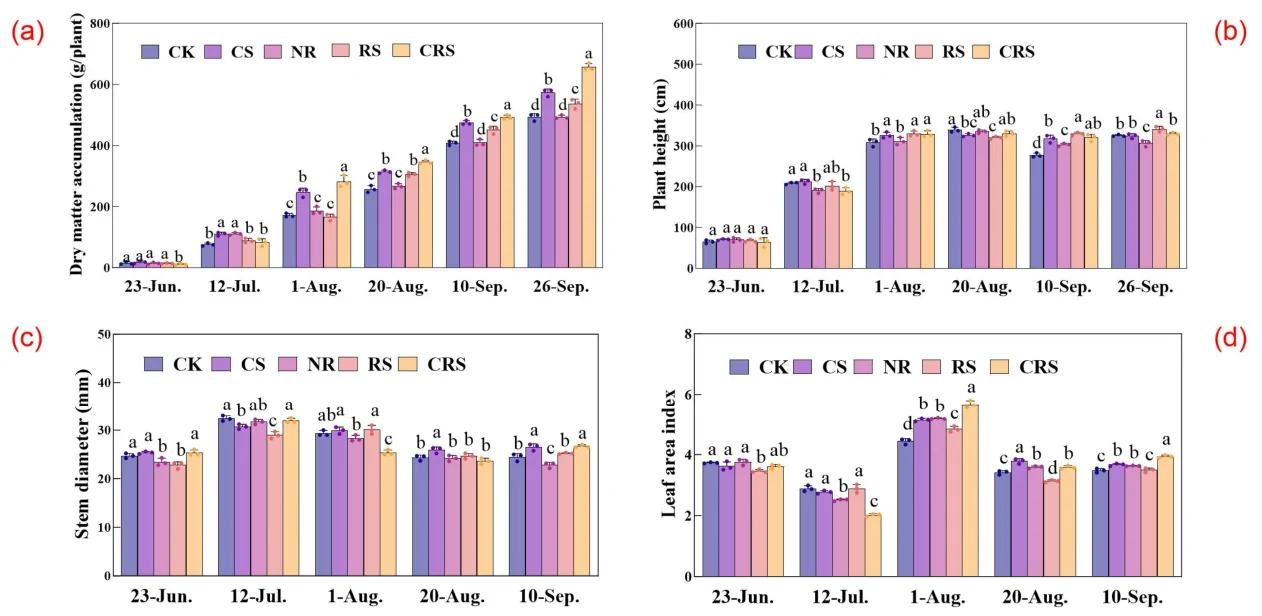
Effects of different tillage methods on dry matter accumulation (**a**), plant height (**b**), stem diameter (**c**), and leaf area index of maize (**d**). Different lowercase letters indicate significant differences among treatments based on Duncan’s multiple range test at *p* < 0.05. Error bars represent standard deviation (*n* = 3).

Different tillage practices affected maize dry matter accumulation, plant height, stem diameter, and leaf area index (Figure 1). Dry matter accumulation increased continuously throughout the growing season (Figure 1a), and differences among treatments became more evident at later growth stages. On 26 September, the CRS treatment showed the highest dry matter accumulation of 656.67 g plant^−1^, which was significantly higher than the 493.33 g plant^−1^ of CK, while the RS treatment ranked second with 536.67 g plant^−1^. Plant height differed little among treatments at the early stages (Figure 1b). At later stages, RS recorded the greatest plant height of 341.00 cm, followed by CRS with 329.33 cm, although overall differences among treatments were limited. Stem diameter reached a maximum at the middle growth stage and then remained stable (Figure 1c), with no significant differences among treatments. Leaf area index first increased and then declined (Figure 1d), reaching a peak on 1 August. The CRS treatment showed the highest leaf area index of 5.66, which was significantly higher than those of the other treatments (*p* < 0.05), and it remained relatively high at later stages with a value of 3.93. Overall, CRS had the highest dry matter accumulation and leaf area index, whereas RS had the greatest plant height at later stages.

### 3.2. Grain Yield and Yield Components

**Figure 2:**
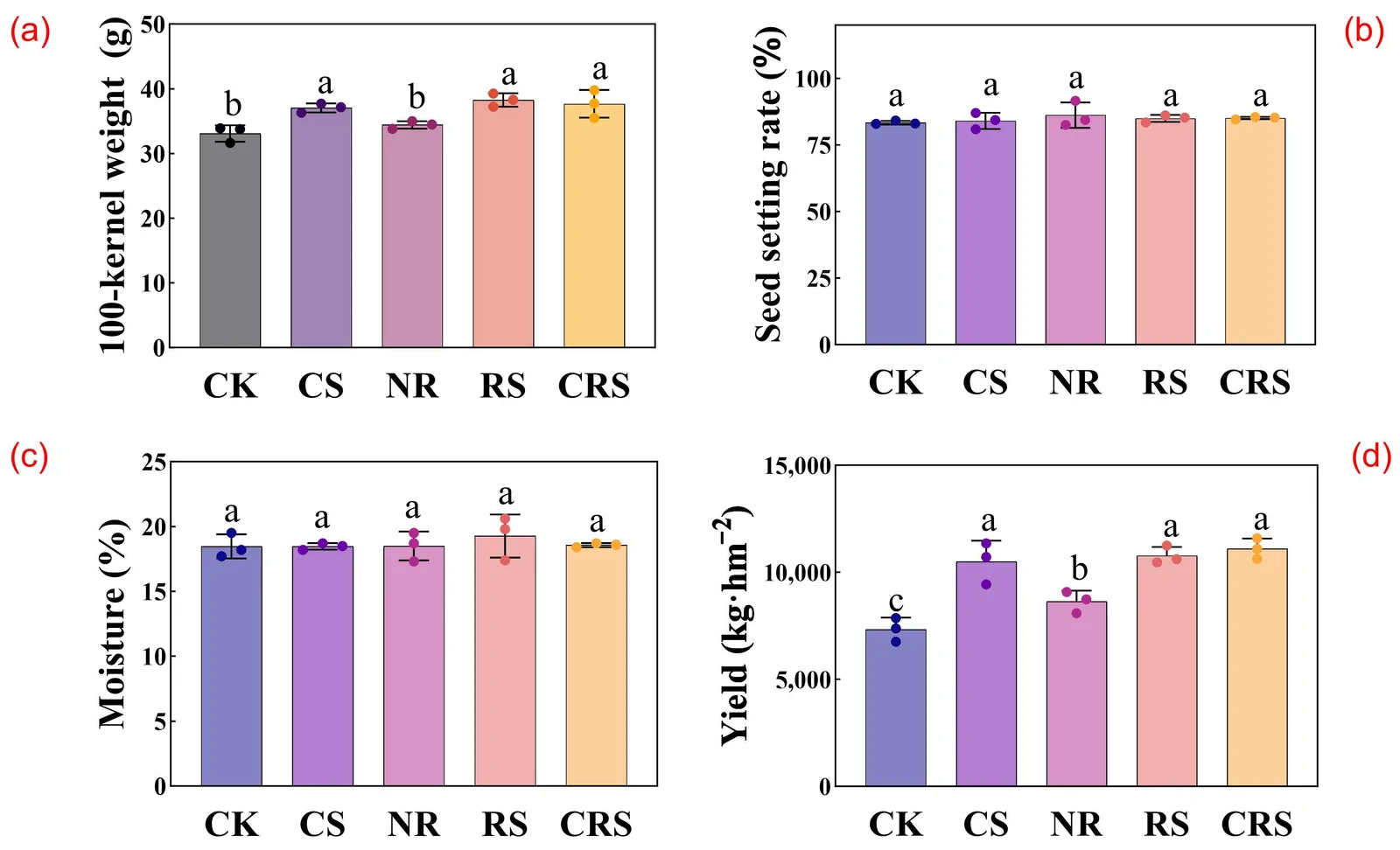
Effects of different tillage methods on 100-grain weight (**a**), seed setting rate (**b**), grain moisture content (**c**), and grain yield (**d**) of maize. Different lowercase letters indicate significant differences among treatments based on Duncan’s multiple range test at *p* < 0.05. Error bars represent standard deviation (*n* = 3).

The 100-grain weight of maize varied among treatments (Figure 2a). Compared with CK, the CS, RS, and CRS treatments significantly increased 100-grain weight by 11.93%, 14.99%, and 13.80%, respectively. No significant difference in 100-grain weight was observed between CK and NR. Seed-setting rate remained relatively high across all treatments (Figure 2b), with no significant differences among treatments. As shown in Figure 2c, the RS treatment had the highest grain moisture content, although differences among treatments were not significant. Grain yield differed among treatments (Figure 2d). Compared with CK, grain yield was significantly higher under the other treatments. Specifically, yields under CS, RS, and CRS increased by 43.28%, 39.91%, and 51.49%, respectively. Overall, CK showed the lowest performance, whereas no significant treatment effect was detected for seed-setting rate.

### 3.3. Variations in Soil Respiration Under Different Tillage Practices

**Figure 3:**
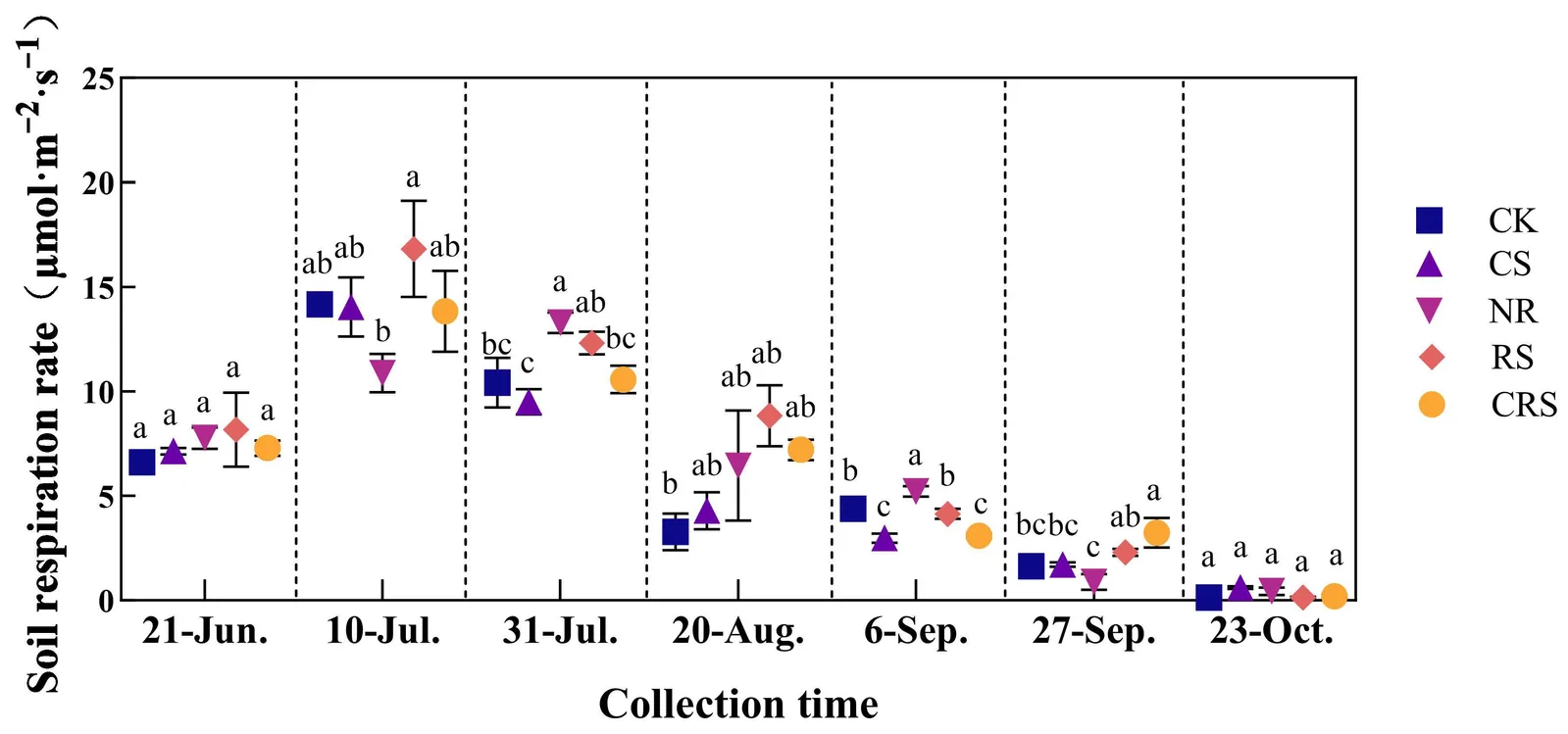
Soil respiration rates under different tillage treatments during the growing season. Error bars represent standard deviation (*n* = 3). At each sampling date, different lowercase letters indicate significant differences among treatments according to Duncan’s multiple range test (*p* < 0.05).

Tillage practices significantly affected the seasonal dynamics of soil respiration (Figure 3). All treatments showed a similar temporal pattern: soil respiration was relatively low in June, increased rapidly, reached a peak in July, and then gradually declined until the end of sampling in October. In late June, soil respiration rates continued to rise. By mid-July, all treatments except NR had reached their peak values, whereas NR peaked at the end of July with a flux of 13.30 μmol m^−2^ s^−1^. The RS treatment reached its peak on 10 July, with a flux of 16.83 μmol m^−2^ s^−1^, which was 18.77–54.69% higher than those of the other treatments. Differences among treatments were most pronounced during the peak growing season from late July to August.

### 3.4. Contribution of Root Respiration to Total Soil Respiration

**Figure 4:**
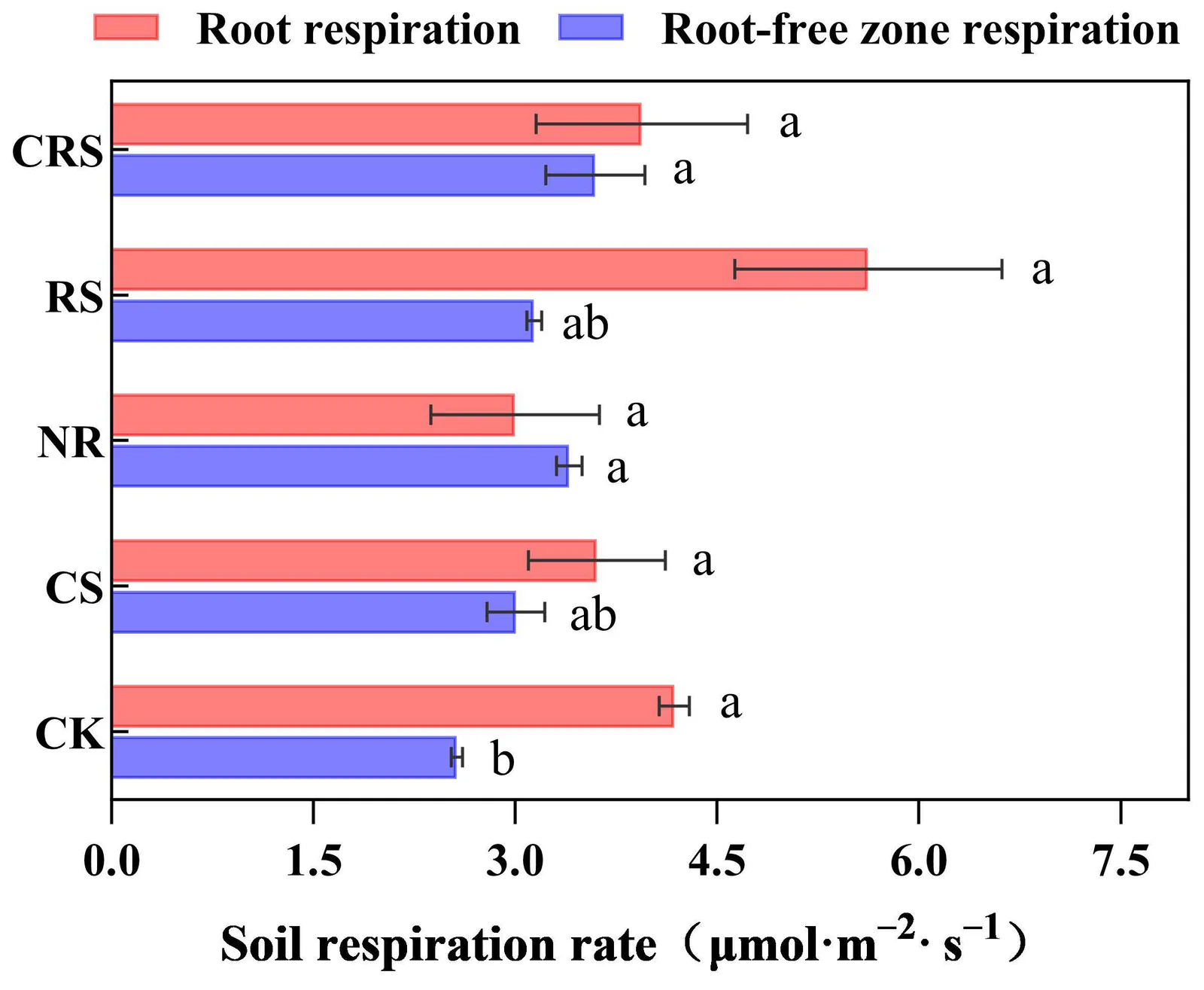
Root respiration and root-free zone respiration under different tillage treatments. Error bars represent standard deviation (*n* = 3). Within the same respiration component, different lowercase letters indicate significant differences among treatments according to Duncan’s multiple range test (*p* < 0.05). The contributions of root respiration to total soil respiration were 61.97%, 54.55%, 46.84%, 64.16%, and 52.30% under CK, CS, NR, RS, and CRS, respectively.

Root respiration and root-free-zone respiration rates differed significantly among tillage treatments (Figure 4). Among the treatments, RS had the highest root respiration rate (5.63 μmol m^−2^ s^−1^), which was significantly higher than those of the other treatments. The root respiration rates under CRS, CK, CS, and NR were 3.94, 4.18, 3.61, and 3.00 μmol m^−2^ s^−1^, respectively. For the contribution of root respiration to total soil respiration, CK and RS showed relatively high values, reaching 61.97% and 64.16%, respectively. However, the contribution of root respiration did not differ significantly among treatments (*p* > 0.05).

### 3.5. Interactive Effects of Soil Temperature and Moisture on Soil Respiration

**Figure 5:**
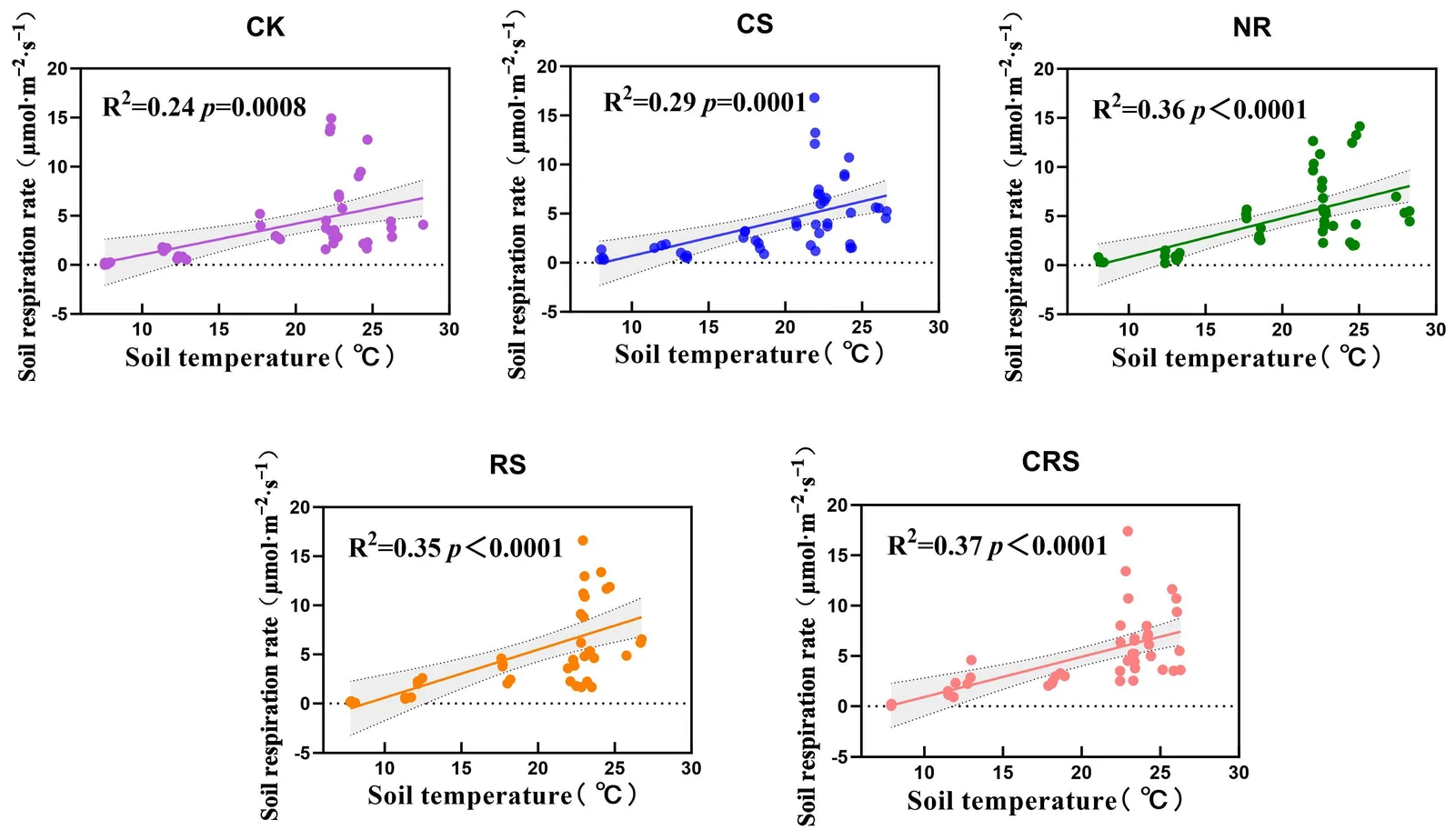
Interactive effects between soil temperature and soil respiration under different tillage transition practices. Solid lines represent fitted linear regressions, and dashed lines indicate the upper and lower 95% confidence intervals.

Under different tillage transition practices, soil temperature was positively and highly significantly correlated with soil respiration rate (Figure 5). The fitted equations for all treatments were highly significant (*p* < 0.001). Among the treatments, CRS showed the highest coefficient of determination (*R*^2^ = 0.37, *p* < 0.0001), followed by NR (*R*^2^ = 0.36, *p* < 0.0001) and RS (*R*^2^ = 0.35, *p* < 0.0001). Lower *R*^2^ values were observed for CS (*R*^2^ = 0.29, *p* = 0.0001) and CK (*R*^2^ = 0.24, *p* = 0.0008).

As summarized in Table 3, the temperature sensitivity coefficient (Q_10_) varied among treatments. The Q_10_ values followed the order NR > CS > RS > CK > CRS, with values of 5.00, 4.38, 4.23, 3.77, and 2.97, respectively. For the basal respiration rate (a), CRS had the highest value of 0.6449, whereas NR had the lowest value of 0.2460. The temperature response coefficient (b) showed a pattern similar to that of Q_10_, with the highest value of 0.1609 in NR and the lowest value of 0.1088 in CRS.

**Figure 6:**
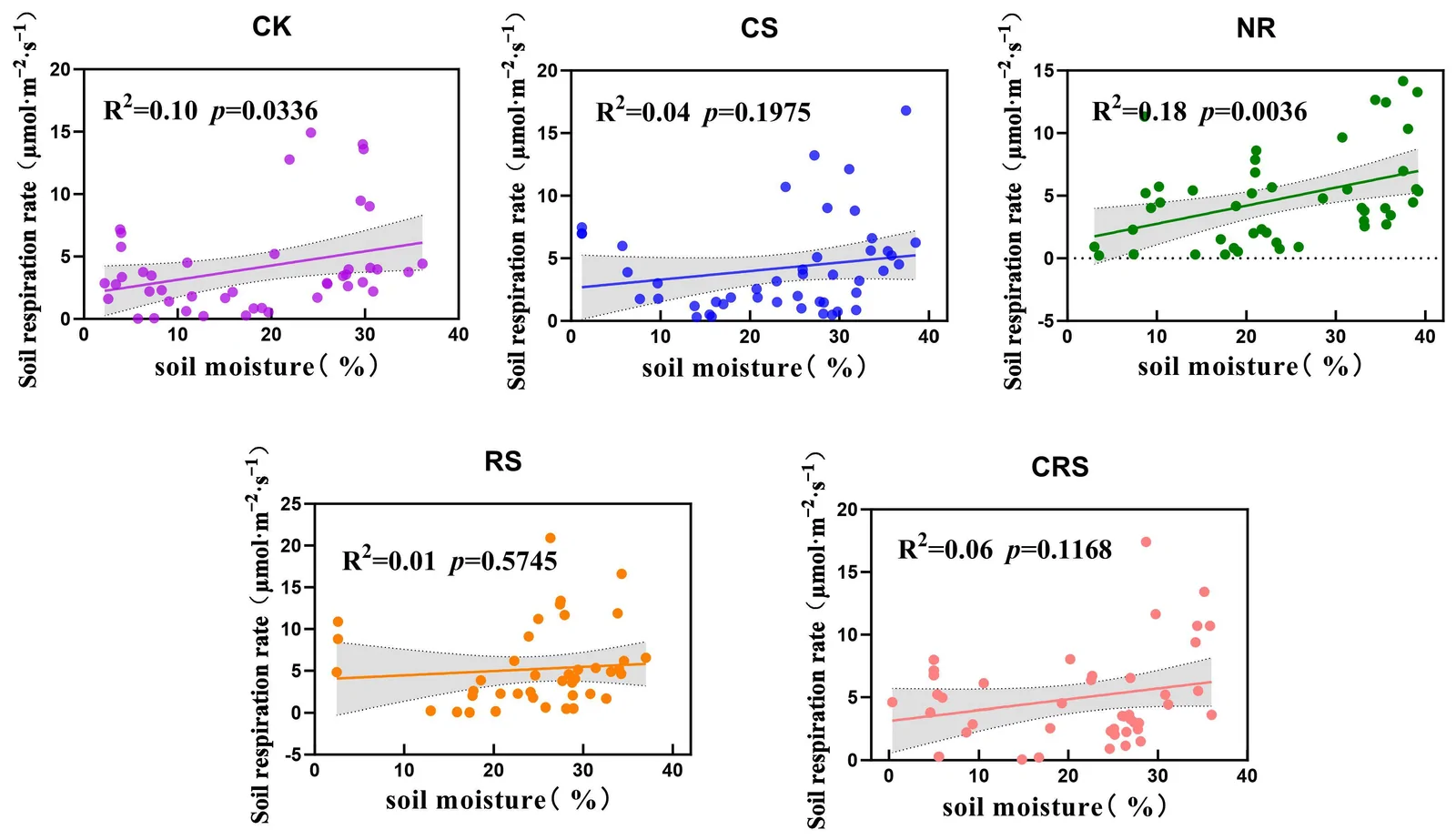
Interactive effects between soil moisture and soil respiration under different tillage transition practices. Solid lines represent fitted linear regressions, and dashed lines indicate the upper and lower 95% confidence intervals.

Under different tillage transition practices, the relationship between soil moisture and soil respiration rate differed among treatments (Figure 6). In the CK treatment, soil respiration rate increased significantly with increasing soil moisture (*R*^2^ = 0.10, *p* = 0.0336). In the NR treatment, soil respiration rate was also significantly and positively correlated with soil moisture (*R*^2^ = 0.18, *p* = 0.0036). The CS treatment showed a positive trend, but the relationship was not significant (*R*^2^ = 0.04, *p* = 0.1975). In the RS and CRS treatments, no significant linear relationship was detected between soil respiration rate and soil moisture (*R*^2^ = 0.01, *p* = 0.5745; *R*^2^ = 0.06, *p* = 0.1168, respectively). Overall, significant positive correlations between soil moisture and soil respiration rate were observed in CK and NR, whereas no significant relationships were found in CS, RS, or CRS.

### 3.6. Effects of Different Treatments on Leaf Photosynthetic Characteristics

**Figure 7:**
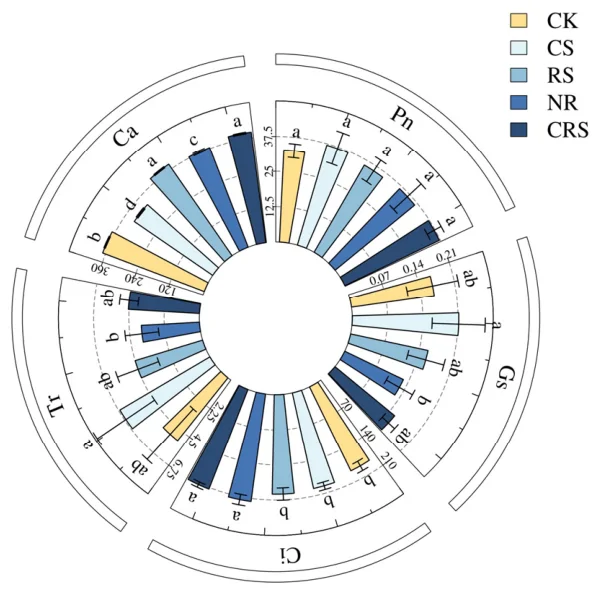
Photosynthetic characteristics of maize leaves under different treatments. The radial bar chart shows the net photosynthetic rate (Pn), stomatal conductance (Gs), intercellular CO_2_ concentration (Ci), canopy CO_2_ concentration (Ca), and transpiration rate (Tr) at the silking stage. Different lowercase letters indicate significant differences among treatments based on Duncan’s multiple range test at *p* < 0.05. Error bars represent standard deviation (*n* = 5). Abbreviations: Pn, net photosynthetic rate (μmol m^−2^ s^−1^); Gs, stomatal conductance (mol m^−2^ s^−1^); Ci, intercellular CO_2_ concentration (μmol mol^−1^); Ca, canopy CO_2_ concentration (μmol mol^−1^); Tr, transpiration rate (mmol m^−2^ s^−1^).

Different tillage treatments significantly affected the photosynthetic characteristics of maize leaves (Figure 7). Compared with CK, the net photosynthetic rate (Pn) under CS reached 36.97 μmol m^−2^ s^−1^, representing an increase of 13.17%. The CRS, RS, and NR treatments increased Pn by 13.67%, 11.45%, and 8.97%, respectively. The highest stomatal conductance (Gs) was observed in the CS treatment, with a value of 0.211 mol m^−2^ s^−1^, which was 27.11% higher than that of CK. The highest intercellular CO_2_ concentration (Ci) was recorded in the RS treatment at 216.07 μmol mol^−1^, which was 15.76% higher than that of CK, followed by the CRS treatment at 213.31 μmol mol^−1^, which was 14.28% higher than CK. The highest transpiration rate (Tr) was also observed in the CS treatment, reaching 8.84 mmol m^−2^ s^−1^, which was 8.60% higher than that of CK. The highest canopy CO_2_ concentration (Ca) was found in the CRS treatment at 379.78 μmol mol^−1^, which was 2.65% higher than that of CK. Overall, CS had the highest Gs and Tr, RS had the highest Ci, and CRS had the highest Ca.

### 3.7. Correlations Among Soil, Gas Flux, and Plant Parameters

**Figure 8:**
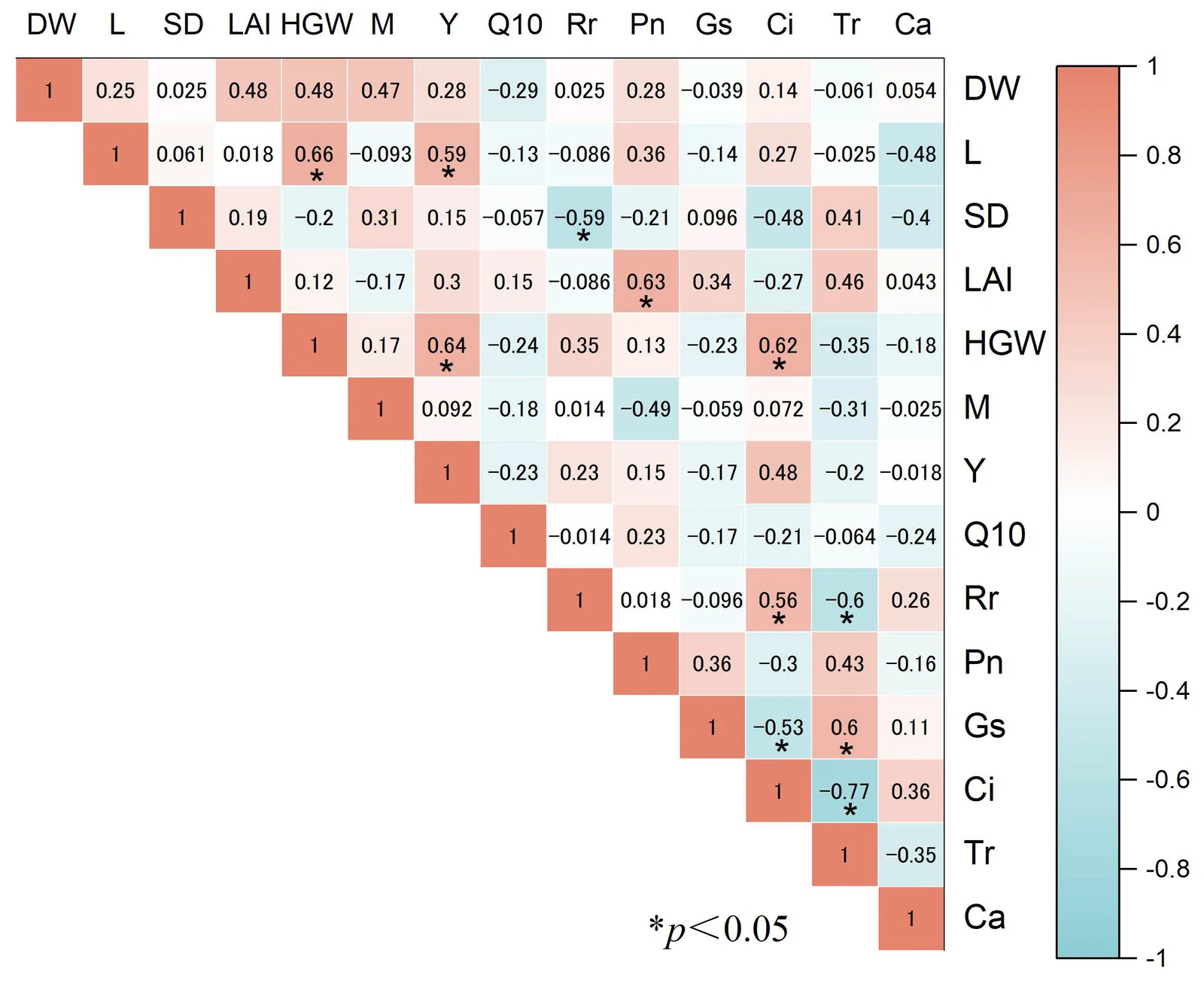
Correlation analysis of maize growth, yield, and yield components with soil respiration indicators and photosynthetic characteristics. Note: DW, dry matter; L, plant height; SD, stem diameter; LAI, leaf area index; HGW, 100-grain weight; M, moisture; Y, yield; Q_10_, soil respiration temperature sensitivity; Rr, root zone respiration rate; Pn, net photosynthetic rate; Gs, stomatal conductance; Ci, intercellular CO_2_ concentration; Ca, canopy CO_2_ concentration; Tr, transpiration rate. Values are Spearman correlation coefficients. * indicates significant correlation at *p* < 0.05. Red indicates positive correlations, and blue indicates negative correlations.

Correlation analysis showed that maize growth, yield, and yield components were associated, to varying degrees, with soil respiration indicators and photosynthetic characteristics (Figure 8). Dry matter accumulation (DW) was strongly and positively correlated with plant height (L), leaf area index (LAI), 100-grain weight (HGW), and yield (Y). Plant height (L) was significantly correlated with LAI and Y (*r* = 0.66^*^ and 0.59^*^, respectively), and HGW was also significantly and positively correlated with Y (*r* = 0.64^*^). This indicates that morphological development and grain filling are key foundations for yield formation.

Among the photosynthetic characteristics, net photosynthetic rate (Pn) was positively correlated with stomatal conductance (Gs) (*r* = 0.36) and negatively correlated with intercellular CO_2_ concentration (Ci) (*r* = −0.30). Gs was significantly negatively correlated with Ci (*r* = −0.53^*^). In addition, Ci was significantly negatively correlated with transpiration rate (Tr) (*r* = −0.77^*^). This indicates that intercellular CO_2_ concentration is closely related to water loss and that a high transpiration rate may accelerate the consumption of intercellular CO_2_, thereby reducing its concentration.

Among the soil respiration indicators, root-zone respiration rate (Rr) was significantly positively correlated with Ci (*r* = 0.56^*^) and significantly negatively correlated with Tr (*r* = −0.60^*^). The results suggest that root zone metabolic activity may participate in physiological regulation by affecting intercellular CO_2_ supply and transpiration.

## 4. Discussion

### 4.1. Mechanisms Underlying the Effects of Tillage Transition on Soil Respiration Components

Soil respiration in ecosystems ranging from forests to grasslands and wetlands typically follows a unimodal pattern, a trend also commonly observed in agricultural soils [20,21]. Consistent with this pattern, the seasonal variation in soil respiration under the five tillage practices during the key growth stages of maize followed a unimodal trend, with an initial increase followed by a decline. The high respiration under RS can be attributed to increased soil porosity and residue incorporation, which accelerated CO_2_ release and root respiration. In addition, the higher contribution of root respiration under the RS treatment suggests that repeated rotary tillage improved soil aeration and porosity, thereby facilitating root respiration and nutrient uptake [22].

The transition from mulching to rotary tillage promoted CO_2_ emissions following tillage, clearly indicating a “priming effect” induced by soil disturbance through aggregate disruption and the exposure of protected organic matter. This phenomenon highlights the immediate carbon cost associated with tillage transition and should be taken into account when evaluating short-term management changes [23,24]. Notably, although the CRS treatment showed the highest basal respiration rate (a-value), its contribution from root respiration was the lowest. This suggests that when a straw-enriched no-till system is disturbed by rotary tillage, the exposure of large amounts of labile carbon substrates may dominate soil respiration in the short term, thereby temporarily suppressing or masking the contribution of root respiration [25].

### 4.2. Regulation of Temperature Sensitivity (Q_10_) of Soil Respiration by Tillage Practices

Tillage transition significantly altered Q_10_, which ranged from 2.97 to 5.00 across treatments. Notably, the NR treatment, which involved a shift from conventional tillage to rotary tillage, exhibited the highest Q_10_ value, suggesting that this system may contain a relatively large pool of temperature-sensitive substrates. The transition likely broke up soil aggregates, mobilizing previously protected organic matter and making it accessible to microbial decomposition; concurrent warming further accelerated microbial metabolism, collectively driving the observed elevation in short-term Q_10_ [26].

In contrast, the Q_10_ value of the CRS treatment (mulching–rotary tillage) was significantly lower, indicating that respiration in this system was less constrained by temperature fluctuations. This attenuated temperature response may arise from the transition-induced stimulation: fresh, readily decomposable carbon from mulch entering suddenly can strongly boost short-term microbial activity, making respiration more substrate-driven than temperature-driven [27,28]. The differences in Q_10_ among treatments further reveal an important interaction between tillage practices and climate feedback. Treatments involving greater soil disturbance, such as CRS, generally exhibited lower Q_10_ values. This pattern can be interpreted within the framework of the carbon quality–temperature hypothesis: frequent disturbance accelerates the turnover of the most labile carbon fractions, increases the proportion of relatively recalcitrant components in the remaining organic matter pool, and thereby reduces the sensitivity of soil respiration to temperature changes [29]. Accordingly, agricultural systems subjected to high-intensity tillage may show a weaker respiratory response to future climate warming. This potential buffering effect may also involve changes in the composition and stability of soil organic matter, although whether it results in a long-term carbon cost still requires further investigation. By contrast, less disturbed tillage systems, such as NR and CS, had higher Q_10_ values, indicating that their soil respiration was more sensitive to temperature changes while also suggesting greater potential for carbon retention under stable conditions. These findings are important for the construction of models describing soil carbon–climate feedback in agricultural ecosystems.

Soil moisture was also significantly related to respiration under specific treatments. The relationships observed under CK and NR indicate that moisture was a key factor regulating respiration in these systems and that an optimal moisture range may exist. However, no significant correlation between soil respiration and soil moisture was found under CS, RS, or CRS. This suggests that temperature or substrate availability may become the more critical limiting factors under these tillage transitions. These findings are consistent with the multiple-limitation conceptual framework involving moisture, temperature, and substrate proposed by Marleen et al. [30].

### 4.3. Synergy Between Soil Carbon Cycling and Crop Productivity

This study integrated the analysis of soil respiration processes with crop growth indicators, providing a new perspective for evaluating the overall ecological effects of tillage management. The RS treatment, which combined continuous rotary tillage with straw incorporation, achieved relatively high crop yield and biomass while maintaining elevated soil respiration, particularly root respiration. This suggests that the RS system may have established a virtuous cycle characterized by high carbon input through photosynthetic products and efficient carbon turnover, rather than simply representing a pathway of net carbon loss. This “high input–high output” pattern may be ecologically sustainable because it enhances both productivity and system vitality [31].

Two complementary processes may help explain the observed associations between soil respiration and maize yield. First, enhanced soil respiration may elevate canopy CO_2_ concentrations, thereby potentially stimulating photosynthetic carbon assimilation. However, the results of this study support such a relationship only as an association rather than as a definitive mechanism. Second, the mechanical loosening caused by rotary tillage enhances gas exchange and water infiltration in the soil profile, which supports more vigorous root development and ultimately boosts aboveground biomass accumulation [32]. Consistent with this interpretation, correlation analysis showed that leaf area index was significantly positively correlated with yield, and net photosynthetic rate was also positively correlated with yield. In contrast, although NR stimulated total respiration, it did not produce a synchronous increase in yield, indicating lower carbon utilization efficiency. This study also indicates that although CRS maintained a relatively high yield, the high-intensity tillage transitions involved in this system, especially under straw-rich conditions, may lead to substantial short-term carbon loss. By comparison, CS and RS were closer to the desired tillage objectives.

The results of this study further highlight the importance of farming-system stability in coordinating soil carbon sequestration and agricultural productivity. Although continuous single tillage patterns, such as CS or RS, can optimize the soil environment in specific ways, for example, by improving water and thermal conditions or increasing porosity, long-term implementation may also lead to problems such as degradation of soil physical structure, aggregate disruption, and soil compaction. Conversely, this study confirms that although short-term tillage transitions implemented to meet immediate needs, such as CRS, may solve current problems, they can also cause substantial carbon loss and ecosystem disturbance. Therefore, from the perspective of ecological sustainability, we suggest a medium-term, stable, and dynamic management strategy: after implementing a conservation tillage model such as straw mulching for 2 to 3 years, the need for appropriate tillage adjustment should be assessed according to actual field conditions. Such a strategy may avoid the potential drawbacks of long-term single tillage practices while keeping carbon losses caused by tillage conversion infrequent and within an acceptable range. Based on the findings of this study, practical recommendations for local farmers in the semi-arid region of western Jilin Province include avoiding straw burning and adopting minimum-disturbance regimes with surface residue retention to preserve soil structure, conserve moisture, and curb erosive losses. A transitional approach combining residue incorporation with rotary tillage may facilitate the shift from conventional to conservation tillage, while long-term adoption of conservation tillage should be encouraged to enhance soil carbon sequestration and promote sustainable agricultural development.

## 5. Conclusions

The results of this study indicate that the transition in tillage practices significantly altered soil respiration rates, the composition of respiration components, and crop growth performance. Continuous rotary tillage supported greater aboveground biomass accumulation by enhancing root-dominated respiration and improving soil aeration conditions. Differences in the temperature sensitivity of soil respiration were also observed among tillage treatments: respiration under the mulching–rotary tillage (CRS) treatment was more strongly regulated by substrate availability, whereas respiration under mulching–mulching (CS) was more sensitive to temperature changes. From the perspective of production and carbon management, the CS treatment maintained stable grain yield with minimal carbon cost; the CRS treatment achieved the highest yield performance; and continuous rotary tillage was more favorable for root-driven respiration and aboveground biomass accumulation. Overall, different tillage transition pathways presented differentiated trade-off patterns among respiration, carbon turnover, and harvestable yield. From a practical standpoint, it is recommended to implement no-tillage with surface residue retention for at least two growing seasons to allow the soil carbon pool to stabilize before further management adjustments, thereby optimizing the synergy between yield and soil health. It should be noted that this study covered only the 2023–2024 tillage transition period, and the results may not fully capture the long-term dynamics of soil respiration and carbon cycling following tillage conversion. Future multi-year field trials under diverse climatic and soil conditions are needed to further verify the long-term effects of tillage transitions on soil respiration and carbon sequestration.

## Figures and Tables

**Table 1:** Basic physical and chemical properties of the test site.

Parameter	Available Nitrogen (mg/kg)	Available Phosphorus (mg/kg)	Available Potassium (mg/kg)	Organic Matter (%)	pH	Apparent Electrical Conductivity (mS/m)
Value	100.16	14.48	105.25	3.04	6.82	84.47

**Table 2:** Experimental treatment design.

Treatment Combination (2023–2024)	Treatment Code	Collar Position
Conventional–Conventional (Control)	CK	Non-root zone
		Root zone
Mulching–Mulching	CS	Non-root zone
		Root zone
Conventional–Rotary Tillage	NR	Non-root zone
		Root zone
Rotary Tillage–Rotary Tillage	RS	Non-root zone
		Root zone
Mulching–Rotary Tillage	CRS	Non-root zone
		Root zone

**Table 3:** Effects of different treatments on the temperature sensitivity coefficient (Q_10_).

Treatment	a	b	Q_10_
CK	0.3634	0.1327	3.77
CS	0.2743	0.1477	4.38
NR	0.2460	0.1609	5.00
RS	0.3680	0.1442	4.23
CRS	0.6449	0.1088	2.97

## Data Availability

The data that support the findings of this study are avail-able from the corresponding authors upon reasonable request due to privacy.

## Supplementary Materials

The following supporting information can be downloaded at: https://www.mdpi.com/article/10.3390/plants15203134/s1, Figure S1: Field Views of the Experimental Treatments and Operations.; Figure S2: Field Photographs of the Relevant Indicator Measurements.
