# Supplementary material for: Synergistic Effects of Maize Growth and Soil Respiration on Yield Under Tillage Regime Conversion
Source: Plants (Basel). 2026 Oct 14;15(20):3134. doi: 10.3390/plants15203134 (PMC13708725; doi:10.3390/plants15203134)
Supplement: Supplementary file 1 [file plants-15-03134-s001.zip › Supplementary Material-Plants.pdf]

Supplementary Material

Title： Synergistic Effects of Maize Growth and Soil Respiration on Yield under Tillage Regime Conversion

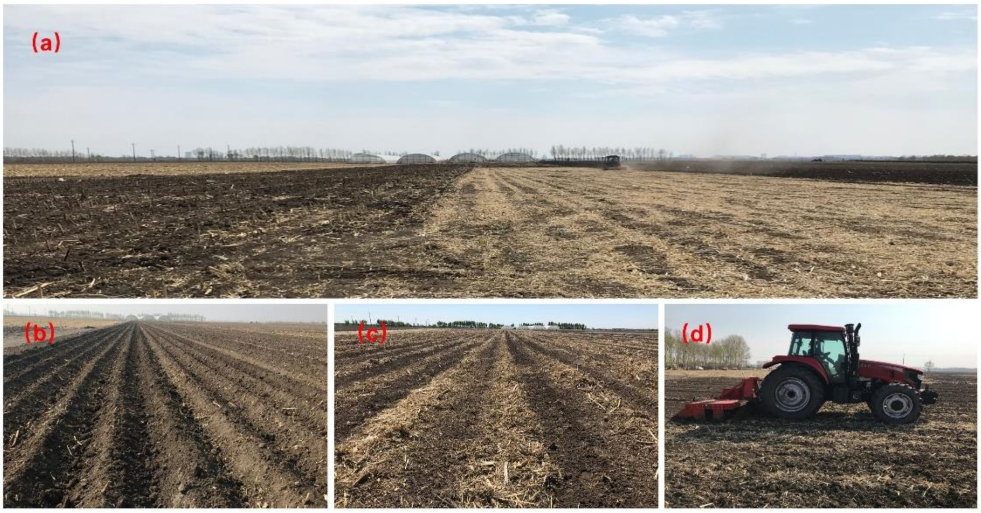

Figure S1. Field views of the experimental treatments and operations. (a) Comparison between conventional tillage without straw (left) and no-till with straw (right); (b) Rotary tillage and ridging without straw; (c) Rotary tillage with straw return; (d) Straw crusher in operation.

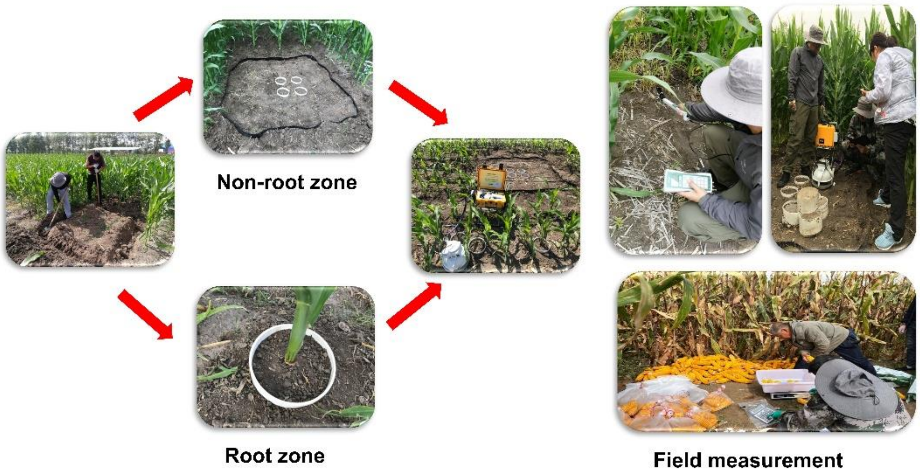

Figure S2. Field photographs of the relevant indicator measurements.
